# The Golgi Apparatus of Neocortical Glial Cells During Hibernation in the Syrian Hamster

**DOI:** 10.3389/fnana.2019.00092

**Published:** 2019-11-19

**Authors:** Gonzalo León-Espinosa, Javier DeFelipe, Alberto Muñoz

**Affiliations:** ^1^Laboratorio Cajal de Circuitos Corticales (CTB), Universidad Politécnica de Madrid, Madrid, Spain; ^2^Departamento de Química y Bioquímica, Facultad de Farmacia, CEU San Pablo University, CEU Universities, Madrid, Spain; ^3^Instituto Cajal, CSIC, Madrid, Spain; ^4^Centro de Investigación Biomédica en Red de Enfermedades Neurodegenerativas, Madrid, Spain; ^5^Departamento de Biología Celular, Universidad Complutense de Madrid, Madrid, Spain

**Keywords:** microglia, astrocytes, oligodendrocytes, torpor, GM130, Golgin84, MG160

## Abstract

Hibernating mammals undergo torpor periods characterized by a general decrease in body temperature, metabolic rate, and brain activity accompanied by complex adaptive brain changes that appear to protect the brain from extreme conditions of hypoxia and low temperatures. These processes are accompanied by morphological and neurochemical changes in the brain including those in cortical neurons such as the fragmentation and reduction of the Golgi apparatus (GA), which both reverse a few hours after arousal from the torpor state. In the present study, we characterized – by immunofluorescence and confocal microscopy – the GA of cortical astrocytes, oligodendrocytes, and microglial cells in the Syrian hamster, which is a facultative hibernator. We also show that after artificial induction of hibernation, in addition to neurons, the GA of glia in the Syrian hamster undergoes important structural changes, as well as modifications in the intensity of immunostaining and distribution patterns of Golgi structural proteins at different stages of the hibernation cycle.

## Introduction

The Golgi apparatus (GA) is a cytoplasmic organelle involved in key cellular processes of the protein and lipid biosynthetic pathways, including post-translational protein modifications and the sorting and trafficking of proteins and lipids to different cellular destinations. It also plays a role in microtubule cytoskeleton organization and calcium buffering (De Matteis and Luini, 2008; Wilson et al., 2011; Rios, 2014). In mammalian cells, the GA is made up of interconnected stacks of flattened cisternae that form a membrane network, termed the Golgi ribbon, which is linked by regions of tubular membranes that connect to the endoplasmic reticulum (ER) (Wei and Seemann, 2010). Each Golgi stack can be classified into three compartments that can be identified according to the enrichment with different GA structural proteins: (1) the *cis*-Golgi network, which is the closest to the ER and is structurally maintained by protein complexes mostly made up of the 65 kD Golgi reassembly stacking protein (GRASP65) and the 130kD *cis*-Golgi matrix protein or GM130 (Nakamura et al., 1995); (2) the medial cisternae where a 160kDa membrane sialoglycoprotein (MG160) is involved in the regulation of growth factor signaling pathways (Gonatas et al., 1995); and (3) the *trans*-Golgi network (TGN), which is located toward the plasma membrane where Golgin84, a rab protein, is involved in the assembly and maintenance of the Golgi ribbon (Diao et al., 2003).

The GA is highly dynamic in brain cells and becomes fragmented in a variety of pathological conditions such as Alzheimer’s disease, amyotrophic lateral sclerosis, Creutzfeldt–Jacob disease, multiple system atrophy, Parkinson’s disease, spinocerebellar ataxia type 2, and Niemann-Pick type C (see Antón-Fernández et al., 2017a,b and references therein). In addition, it has been shown that there is structural plasticity of the GA in a variety of biological processes, such as mitotic cell division and aging in different tissues and cell types (Lucocq et al., 1987; Levine et al., 1995; Ledda et al., 2001; Glick, 2002; Fan et al., 2008; Corda et al., 2012).

Hibernation is a natural process characterized by periods of low body temperature (Tb) and metabolic rates that allows many small mammals to survive for prolonged periods without food (Ruf and Geiser, 2015). One of these species, the Syrian hamster (*Mesocricetus auratus*), hibernates with multiday periods of torpor when Tb and metabolism are reduced to a minimum, interrupted by periodic arousals and brief normothermic periods (Bartness and Wade, 1985; Oklejewicz et al., 2001).

Previous studies have demonstrated that the state of torpor is accompanied by a variety of complex adaptive and reversible brain changes that appear to protect the brain from hypoxia and low Tb. These brain changes during torpor include a decrease in adult neurogenesis; alterations in dendritic trees including dendritic spine retraction; a decrease in synaptic connections; the fragmentation and reduction of the neuronal GA; and an increase in length of the axon initial segment (Popov and Bocharova, 1992; Popov et al., 1992, 2007, 2011; Von Der Ohe et al., 2006, 2007; Antón-Fernández et al., 2015; Bullmann et al., 2016; Leon-Espinosa et al., 2016, 2018).

Interestingly, during torpor bouts, the GA of neocortical and hippocampal neurons undergo a reversible fragmentation accompanied by a reduction in the surface area and volume of the GA elements, as well as alterations in the expression levels and distribution patterns of the GA structural proteins GM130, MG160, and Golgin84 (Antón-Fernández et al., 2015). In addition to these neuronal changes, it has been reported that there is a reduction in the microglial reaction produced by the insertion of microdialysis probes (Zhou et al., 2001) in hibernating arctic ground squirrels. This could be related to the morphological and neurochemical changes that take place in microglial cells during torpor, which show a dystrophic phenotype including an increase in the number and the shortening of Iba-1-ir processes, which show an overall fragmented appearance with thinning at some segments and swellings at others (Leon-Espinosa et al., 2018). Knowledge of the structural organization of the GA of glial cells of the neocortex is fragmentary and limited to pioneering studies with silver staining (Del Rio Hortega, 1919), electron microscopy (Peters et al., 1991), and *in vitro* observations (Lavi et al., 1994). In the present study, using double immunofluorescence and confocal microscopy techniques, we have addressed whether GM130, MG160, and Golgin84 are expressed in the GA of the main three types of glia, namely microglial cells, astrocytes, and oligodendrocytes in the neocortex of the Syrian hamster. Furthermore, we examined whether the GA of these glial cell types undergo morphological adaptations during the hibernation cycle.

## Materials and Methods

In the present study, we used nine male 3-month-old Syrian hamsters (*M. auratus*) that were purchased from Janvier Labs (*Le Genest-Saint-Isle*, France). All experimental procedures were carried out at the animal facility of the Instituto Cajal in Madrid (registration number ES 28079 0000184) and were approved by the institutional Animal Experiment Ethics Committee (PROEX 292/15). The animals had free access to food and water and were kept at 23°C with an 8:16-h light/dark cycle in our animal facility until induction of hibernation (torpor and arousal experimental groups) or until their sacrifice (control animals). After the acclimatization period (4–6 weeks), six out of the nine animals were transferred to a special chamber to artificially induce hibernation. The chamber controls the temperature and illumination, while also allowing the hamsters to be monitored by measuring their locomotor activity. The animals spent 1 week in this special chamber where light was progressively diminished (until complete darkness) as well as temperature (from 23 to 4°C). Torpor was considered to have started when the animals showed periods of inactivity of 24 h, which generally occurred 2–3 months after the chamber temperature had reached 4°C. We considered animals to be torpid only when they had completed three full bouts of torpor (3–4 days of inactivity) before they were sacrificed. As torpor lasts for 3–4 days, we sacrificed the animals on day 2 after torpor started, to make sure the animals were in deep torpor. The arousal experimental group was obtained by removing the torpid animals from the hibernation chamber and inducing their awakening 1 h before they were sacrificed. The temperatures of the animals were checked using an infrared thermometer before perfusion to make sure they were in deep torpor (*n* = 3) or in arousal (*n* = 3). Control animals (*n* = 3) were not transferred to the hibernation chamber. See Antón-Fernández et al. (2015) for further details.

All animals were sacrificed by a lethal intraperitoneal injection of sodium pentobarbital (200 mg/kg) and then perfused intracardially with a saline solution followed by 4% paraformaldehyde in phosphate buffer (PB; 0.1M pH 7.4). The brain of each animal was removed and post-fixed by immersion in the same fixative for 24 h at 4°C. After rinsing in PB, the brains were cut in the coronal plane using a vibratome (Leica VT2100S). Serial sections (50 μm thick) were cryoprotected in 30% sucrose solution in 0.1M PB and stored in ethylene glycol/glycerol at –20°C until they were used.

For immunofluorescence, free-floating sections were rinsed thoroughly in 0.1M PB and incubated for 1 h in 0.1M PB with 0.25% Triton-X100 and 3% BSA (Bovine Serum Albumin; Sigma A4503). Sections were incubated for 48 h at 4°C in the same blocking solution containing double or triple combinations of the antibodies described in Table 1. Those antibodies were directed against the ionized calcium binding adaptor molecule 1 (Iba-1), the glial fibrillary acidic protein (GFAP) (or the intracellular glycoprotein S100β), and the enzyme 2′,3′-Cyclic-nucleotide 3′-phosphodiesterase (CNPase), to label microglial cells, astrocytes and oligodendrocytes respectively, in combination with antibodies that recognize the GA proteins GM130, MG160, and Golgin84.

**TABLE 1:** Summary of primary antibodies and combinations used for immunodetection.

**Target**	**Host species and type**	**Immunogen**	**Dilution factor**	**Supplier**	**Catalog number**
CNPase	Mouse, monoclonal	Purified Full length native human CNPase protein	1:200	Abcam	Ab6319
S100 (β subunit)	Mouse, monoclonal	Purified bovine brain S100 beta preparation	1:1000	Sigma	S2532
Iba-1	Rabbit, polyclonal	Synthetic peptide corresponding to C-terminus of Iba1	1:500	Wako	019-19741
Iba-1/AIF1	Mouse, monoclonal	Synthetic peptide corresponding to human Iba1/AIF1	1:500	Merck	MABN92
Iba-1	Goat, polyclonal	Synthetic peptide corresponding to corresponding to amino acids 135–147 of human Iba-1	1:500	Abcam	ab5076
GFAP	Goat, polyclonal	Synthetic peptide mapping at the C-terminus of human GFAP	1:200	Santa Cruz	sc-6170
Golgi Complex MG160	Rabbit, polyclonal	Synthetic peptide conjugated to KLH, corresponding to a region within the C-terminal amino acids 1150–1179 of human Golgi Complex	1:100	Abcam	ab103439
GM130	Mouse, monoclonal	Synthetic peptide corresponding to amino acids 869–982 from rat GM130	1:50	BD Transduction Laboratories	610822
Golgin 84	Rabbit, polyclonal	Synthetic peptide corresponding amino acids 343–625 mapping within an internal region of human golgin84	1:500	Santa Cruz	sc-134704

After rinsing in PB, the sections were first incubated for 2 h at room temperature in secondary antibodies: biotinylated goat anti-rabbit (1/200) or biotinylated horse anti-mouse (1/200), depending on the primary antibody combination used, in order to amplify the GA marker signal. The sections were then rinsed in 0.1M PB and incubated for 2 h at room temperature in streptavidin-coupled Alexa 488 (1/200; Molecular Probes, Eugene, OR, United States) and Alexa 594-conjugated goat anti-mouse or Alexa 594-conjugated goat anti-rabbit (1/1000; Molecular Probes, Eugene, OR, United States). For triple immunostaining, we used, as secondary antibodies, Alexa 488-donkey anti-goat, Alexa 594-donkey anti-rabbit, and Alexa 647-donkey anti-mouse. In all cases, after rinsing, the sections were also stained with the nuclear stain DAPI (4,6 diamino-2-phenylindole; Sigma, St. Louis, MO, United States). Finally, the sections were mounted in antifade mounting medium (ProLong Gold, Invitrogen) and studied by confocal microscopy (Zeiss, 710).

Confocal image stacks from layers II–III of the somatosensory cortices of the Syrian hamster were recorded at 0.14 μm intervals through separate channels with a 63x oil-immersion lens (NA, 1.40, refraction index, 1.45) at zoom 2.0. Image stacks were taken from 30 whole-brain sections per animal. We used ZEN 2012 software (Zeiss) to construct the maximum intensity projection images from the optical series by combining the images recorded through the different channels (image resolution: 1024 × 1024 pixels; pixel size: 0.066 μm). Adobe Photoshop (CS4) software was used to compose figures.

## Results

In the present study, we qualitatively analyzed the distribution patterns of the GA proteins GM130, MG160, and Golgin84 in microglia, astrocytes, or oligodendrocytes at different stages of the hibernation cycle. Since subtle changes observed in immunocytochemically stained sections are difficult to interpret, we were only looking for large, obvious differences the distribution patterns of GA proteins between different experimental conditions.

### Golgi Apparatus of Microglial Cells During Hibernation

#### Euthermic Hamsters: Resting Phenotype

**FIGURE 1:**
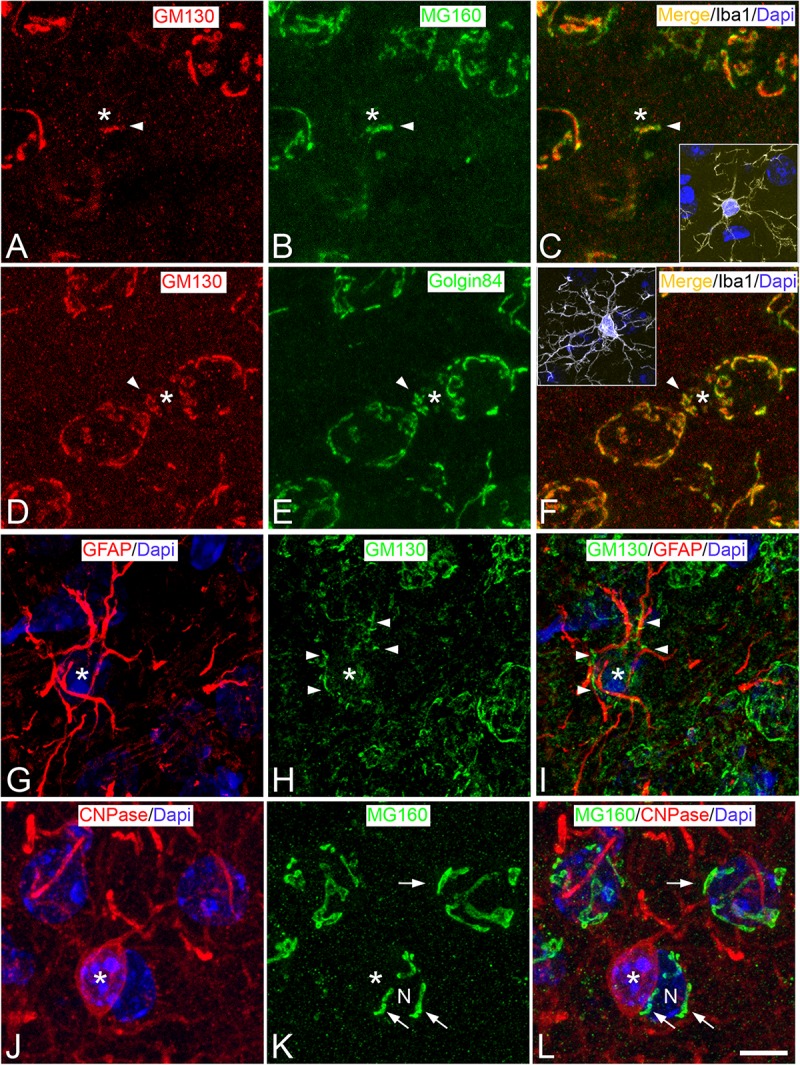
Distribution of GA proteins in neocortical glial cells from euthermic hamsters. **(A–L)** Trios of images taken from neocortical sections immunostained simultaneously for MG160/GM130/Iba-1 **(A–C)**, GM130/Golgin84/Iba-1 **(D–F)**; GFAP/GM130 **(G–I)**, and CNPase/MG160 **(J–L)** showing the distribution of GA proteins in the GA (arrow heads) of microglial **(A–F)**, astrocytes **(G–I)**, and oligodendrocytes **(J–L)** from euthermic hamsters. In microglial cells, note the similar distribution patterns of GM130, MG160, and Golgin84 and the high degree of colocalization. Insets in panels **(C)** and **(F)** show the cell bodies of microglial cells identified by Iba-1 immunostaining (white). Asterisks in panels **(G–I)** and **(J–L)** indicate respectively the cell body of one astrocyte and one oligodendrocyte adjacent to a neuron (N). The arrow indicates the GA of the neuron N. Scale bar shown in panel **(L)** represents 7.2 μm in all panels, and 15.8 μm in the insets.

We first carried out experiments with immunostaining for Iba-1, which is a 17-kDa specific calcium-binding protein that uniformly distributes in the cytoplasm and processes of both activated and resting microglial cells (Ito et al., 1998). In line with previous studies (Cogut et al., 2017; Leon-Espinosa et al., 2018), we found that Iba-1-immunoreactive (-ir) microglial cells in euthermic hamsters had multiple long, thin and highly branched processes typical of the ramified/resting state (inset Figures 1C,F).

**FIGURE 2:**
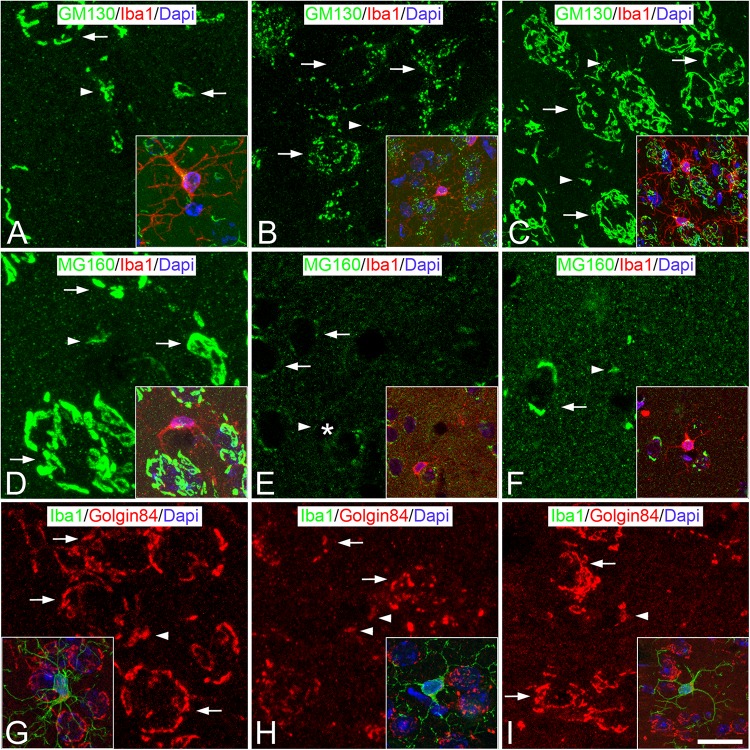
Distribution of GA proteins in neocortical microglial cells throughout the hibernation cycle of the hamster. Images taken from neocortical sections double-immunostained for GM130/Iba-1 **(A–C)**, MG160/Iba-1 **(D–F)** and Golgin84/Iba-1 **(G–I)** showing the distribution of GA proteins in the GA of microglial cells (identified in insets by Iba-1 immunostaining) from euthermic hamsters **(A,D,G)**, hamsters at torpor **(B,E,H),** and arousal **(C,F,I)**. Note the apparent reduction in size and in intensity of MG160 immunostaining of the GA elements of microglial cells during torpor and recovery in arousal. Scale bar shown in panel **(I)** indicates 6.8 μm in all panels, and 15 μm in the insets.

According to pioneering studies, based on silver staining (Del Rio Hortega, 1919) and electron microscopy (Peters et al., 1991), double immunocytochemistry for Iba-1 and GM130 revealed that GA elements of microglial cells were intensely immunostained and grouped, at a perinuclear position at one pole of the cell soma (Figures 1A,C,D,F, 2A). In addition, we found that the GA of microglial cells often partially extend toward one of the cell processes emerging from the cell body, usually the one that is most prominent or thick (Figure 2A). GM130-ir GA elements in this microglial cell process were observed either in continuity or disconnected from the main GA.

Immunostaining for MG160 (Figures 1B, 2D) or Golgin84 (Figures 1E, 2G) revealed that these two proteins are also expressed in the GA of euthermic hamsters and it revealed a similar morphological appearance of the GA to that observed in sections immunostained for GM130. A large degree of MG160/GM130 and GM130/Golgin84 colocalization was found in Iba-1/MG160/GM130 and Iba-1/GM130/Golgin84 triple-immunostained sections (Figures 1A–F).

#### Torpid and Aroused Hamsters: Hibernating Dystrophic Phenotype

The GA of dystrophic microglial cells from torpid hamsters showed morphological alterations, as seen by comparing it with the GA from euthermic microglial cells (Figure 2). During torpor, based on both GM130 and Golgin 84 immunostaining, the GA of microglial cells had a fragmented appearance, with

sparse immunostained punctate, unconnected elements that were distributed at one pole of the cytoplasm. These changes occurred with an apparent reduction in the size of the GA and with no apparent reductions in the intensity of GM130 and Golgin 84 immunostaining (Figures 2A,B,G,H). By contrast, in MG160/Iba-1 double-immunostained sections, in line with the reduction of MG160 observed in neurons during torpor (Antón-Fernández et al., 2015), we found a complete lack of MG160 immunostaining in the cell body of the great majority of the microglial cells (Figure 2E). Furthermore, based on the immunostaining for the three GA markers used, the morphology of the GA of microglial cells partially recovers during arousal, when hamsters awake from torpor, with it resembling the characteristics of the GA in euthermia described above (Figures 2C,F,I).

### Distribution of Golgi Proteins in Protoplasmic Astrocytes

#### Euthermic Hamsters

**FIGURE 3:**
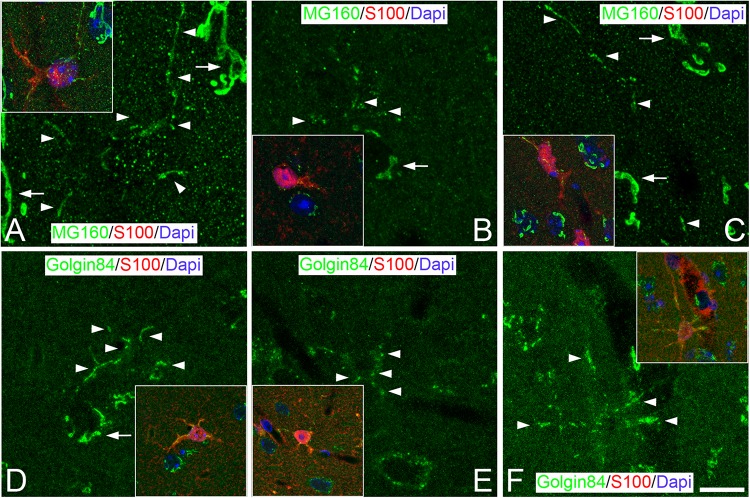
Distribution of GA proteins in neocortical astrocytes throughout the hibernation cycle of the hamster. Images taken from neocortical sections double-immunostained for MG160/S100 **(A–C)** or Golgin 84/S100 **(D–F)** showing the distribution of GA proteins in the GA of astrocytes (identified by S100 immunostaining) from euthermic hamsters **(A,D)**, hamsters at torpor **(B,E)**, and arousal **(C,F)**. Note the apparent reduction in size and in intensity of MG160 immunostaining and the fragmentation of the GA elements of astrocytes during torpor and recovery in arousal. Scale bar shown in F indicates 7.2 μm in all panels, and 15.8 μm in the insets.

We have studied the GA of layer II–III astrocytes by double immunofluorescence using all of the possible combinations of antibodies against the GA proteins GM130, MG160, and Golgin84 with antibodies against GFAP – a brain-specific component of intermediate filaments in the cytoplasm of astrocytes – and S100β to identify astrocytes (Figures 1G–I, 3). Antibodies against GM130 could only be combined with antibodies against GFAP. A limitation when using GFAP immunostaining to analyze the GA of astrocytes is that it intensely labels filament bundles of the astrocyte cytoskeleton without rendering a complete labeling of the astrocyte cytoplasm. This makes it difficult to distinguish which GM130-ir elements are contained within a particular astrocyte and which belong to adjacent cells (Figures 1G–I). Regardless of this limitation, in GM130/GFAP double-immunostained sections from euthermic hamsters, we found that intermingled with GFAP-ir fibrous bundles, there were MG130-ir elements of the GA consisting of round and mainly elongated or tubular elements distributed throughout the cell soma, with such elements extending a considerable distance, along all the cell processes emanating from the soma (Figures 1G–I). This distribution of GA elements was confirmed in sections immunostained for MG160 or Golgin84 (the other two GA protein markers), each of which was in combination with immunostaining for the beta chain of S100 – a calcium binding protein that is mainly found in the cytoplasm of astrocytes and that allows the somata and the processes of astrocytes to be clearly distinguished from adjacent cellular elements. Distribution and morphological features of GA elements similar to those described above for GM130 were found in GM160/S100 and Golgin84/S100 double-immunostained sections from euthermic hamsters (Figures 3A,D, respectively).

#### Torpid and Aroused Hamsters

We next studied, in GM160/S100β or Golgin84/S100β immunostained sections, whether the morphology of the GA of protoplasmic astrocytes undergo plastic changes throughout the different phases of the hibernation cycle (Figure 3). We observed that, during torpor, MG160 staining was reduced in the cytoplasm of astrocytes (Figure 3B). By contrast, in Golgin84/S100 immunostained sections, we found that the expression of Golgin 84 in astrocytic GA elements was preserved during torpor, although these elements showed different morphological properties to those seen in the GA of astrocytes in euthermia. Golgin84 and MG160-ir GA elements had a punctate or fragmented appearance, both in the soma and the processes of astrocytes, with tubular elements only occasionally found (Figures 3B,E). During arousal, MG160 and Golgin84 immunostaining tended to revert, showing immunostained GA elements in astrocytes with the same appearance as in euthermic animals (Figures 3C,F).

### Distribution of Golgi Proteins in Perineuronal Oligodendrocytes

#### Euthermic Hamsters

**FIGURE 4:**
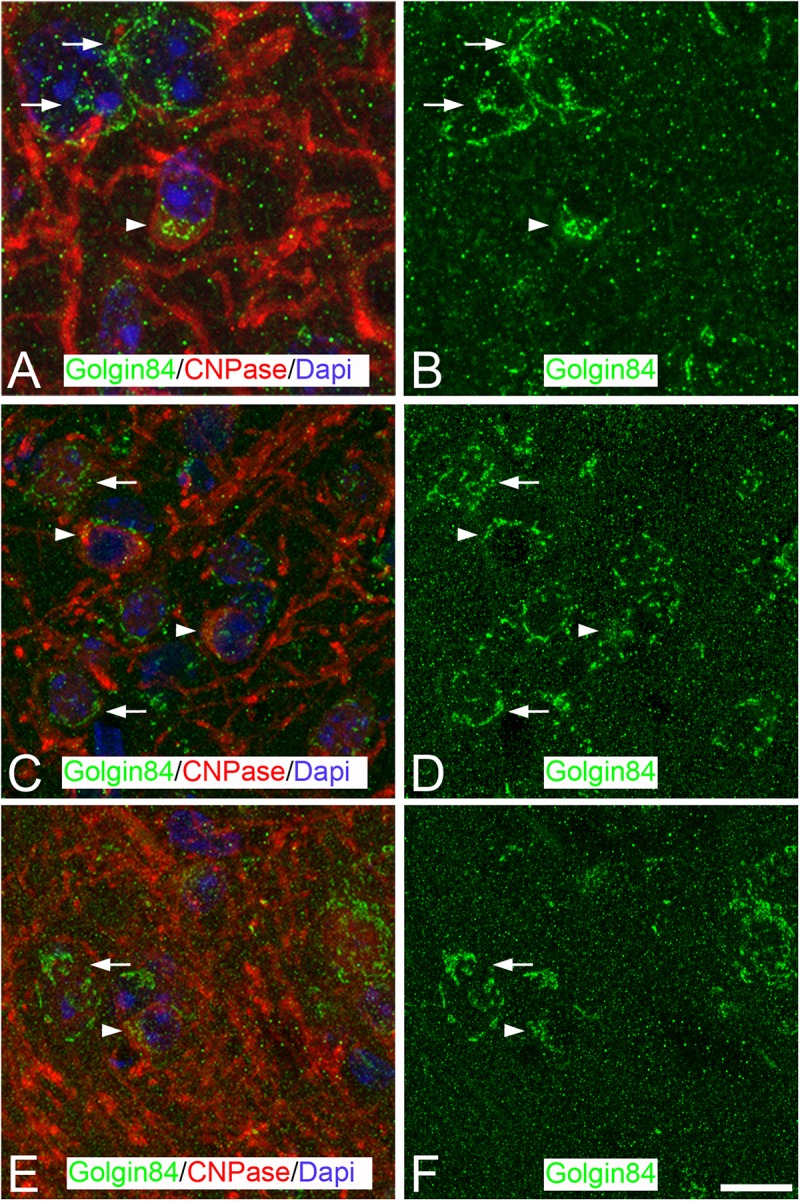
Distribution of GA proteins in neocortical oligodendrocytes throughout the hibernation cycle of the hamster. **(A–F)** Pairs of mages taken from sections of the neocortex double-immunostained for Golgin 84/CNPase showing the distribution of Golgin84 [green in panels **(B,D,F)**] in the GA of oligodendrocytes (identified by CNPase immunostaining) from euthermic hamsters and hamsters at torpor and arousal, respectively. Note the apparent disorganization of the GA elements of oligodendrocytes during torpor and the recovery in arousal. Scale bar shown in panel **(F)** indicates 6.8 μm in all panels.

Basing our approach on previous studies (Trapp et al., 1988), we have used antibodies that recognize CNPase to label oligodendrocytes. This antibody could only be combined with the antibodies for the GA proteins Golgin84 and MG160 (Figures 1J–L, 4). Therefore, the distribution of GM130 in the GA of oligodendrocytes could not be studied. In sections immunostained for CNPase and MG160, we found no immunostaining for MG160 in the cell bodies of oligodendrocytes, indicating a lack of expression of this protein (Figures 1J–L). By contrast, we found Golgin 84-ir elements in the cytoplasm of euthermic hamster oligodendrocytes in CNPase/Golgin84 double-immunostained sections (Figure 4). In line with previous electron microscope descriptions of the GA of oligodendrocytes (Peters et al., 1991), we found that Golgin84-ir elements displayed rounded or tubular shapes and were located, in euthermic hamsters, throughout the perikaryal cytoplasm, although they tended to aggregate at one pole (Figures 4A,B). In addition, in contrast to microglial cells and astrocytes, we did not find GA elements in oligodendrocytes extending into the processes emanating from the cell body (Figures 4A,B).

#### Torpid and Aroused Hamsters

The morphological appearance of the GA of oligodendrocytes is also affected during the hibernation cycle. During torpor, Golgin84-ir elements were often found dispersed throughout the perikaryal cytoplasm showing a sand-like appearance in contrast with observations in euthermic animals (Figures 4C,D). In aroused hamsters, we found a partial recovery of the GA fragmentation that occurred during torpor (Figures 4E,F).

## Discussion

In the present study, using immunocytochemistry and confocal microscopy, we have characterized the distribution and the morphological features of the GA of microglial, astroglial, and oligodendroglial cells of the somatosensory cortex of the Syrian hamster during euthermia (control) conditions, on the basis of expression of the GA structural proteins GM130, MG160, and Golgin84. In addition, we have shown that the GA of glial cells undergo profound but reversible cell type-specific structural changes during the different phases of the hibernation cycle, along with cell-type- and protein-specific modifications in expression levels and distribution patterns of Golgi structural proteins.

### GA of Neocortical Glial Cells During Euthermia

Previous studies from our laboratory showed that the GA of neocortical pyramidal neurons in the Syrian hamster consisted of a ribbon-like network of tubular elements and stacked cisternae that are distributed throughout the cell body around the nucleus and partially extended to the apical dendrite (Antón-Fernández et al., 2015). In the present study, we show that in glial cells, the GA is less prominent than in pyramidal neurons and that there are structural differences between the three types of glial cells analyzed. In ramified/resting microglial cells of euthermic hamsters, the GA consisted of rounded elements mainly located in a perinuclear position at one pole of the cell soma, which is in line with previous studies (Del Rio Hortega, 1919; Peters et al., 1991). In addition, we noted that scarce GA elements often extend a short distance toward just one process, usually the thickest one, radiating from the microglial cell body. As we have not observed GM130-immunostained elements in more than one microglial cell process, these observations suggest the existence of an asymmetry between microglial cell processes regarding the presence of GA elements. In astrocytes, in contrast to microglial cells, GA elements mainly consisted of elongated or tubular elements distributed throughout the cell soma and extending along all the cell processes emanating from the soma. Finally, the GA in oligodendrocytes consisted of rounded or tubular shaped elements distributed throughout the perikaryal cytoplasm, although these elements tended to aggregate at one pole, in agreement with previous electron microscope observations (Peters et al., 1991). However, in contrast to microglial cells and astrocytes, GA elements were never found to extend to the processes emanating from the cell body.

Previous observations in the GA of neocortical and hippocampal pyramidal neurons from euthermic Syrian hamsters showed a differential expression of GM130, MG160, and Golgin84 (Antón-Fernández et al., 2015). The present study extends these observations to glial cells, showing that these three GA proteins are expressed in the GA of microglial cells and astrocytes. As described for neurons (Antón-Fernández et al., 2015), GM130, MG160, and Golgin84 colocalize to a large extent in the GA of microglial cells and astrocytes, but not fully. Further electron microscopy observations of immunocytochemically stained sections would be necessary to analyze – at higher resolution – the extent and organization of the GA microdomains expressing different combinations of GA proteins in neocortical cells. In contrast to microglial cells and astrocytes, in the present work we found that oligodendrocyte GA expresses Golgin 84, but not MG160. Unfortunately, the expression of MG130 in oligodendrocytes could not be tested. The structural and functional consequences of the lack of MG160 expression in the GA of oligodendrocytes would require further research.

### GA of Neocortical Glial Cells During Hibernation

A drop-in energy expenditure is essential for hibernating mammals to overcome the difficulties associated with winter. Energetically demanding cellular processes such as transcription and protein synthesis are severely reduced during torpor bouts and recover fully during the interbout arousal (Rolfe and Brown, 1997; Van Breukelen and Martin, 2002). In the brain, mammalian hibernation involves many plastic responses, in parallel with the cessation of neuronal activity (Strumwasser, 1959a, b,c; South, 1972; Walker et al., 1977; Igelmund and Heinemann, 1995). These changes are thought to prevent neural damage as a consequence of extreme conditions of both hypoxia and low Tb. Hibernation is accompanied by changes in the overall phosphorylation state required for the metabolic energy-saving processes (suppression of metabolism and thermogenesis). In particular, Golgi ribbon linking is regulated by phosphorylation of Golgi stacking proteins. For instance, during mitosis, the GM130 protein is phosphorylated by Cyclin-Dependent Kinase 1, inhibiting binding to p115 and promoting GA fragmentation (Lowe et al., 2000). Moreover, the Golgi reassembly stacking proteins of 55 kDa (GRASP55) and 65Kd (GRASP65) are also phosphorylated during mitosis. This causes Golgi ribbon unlinking and cisternal unstacking, although reassembly occurs upon dephosphorylation (Tang et al., 2012; Huang and Wang, 2017). These data suggest that the protein phosphorylation during hibernation may affect GA stacking proteins as well, resulting in the GA disorganization. Another hypothesis, that may explain the overall GA changes in glial cells during hibernation – is that the temperature drop may affect membrane integrity. In general, lipid metabolism regulation is crucial during hibernation (Lang-Ouellette et al., 2014) but, in particular, organelle lipids undergo a rapidly reversible rearrangement caused by a temperature reduction (Azzam et al., 2000). *In vitro* experiments described significant morphological changes in the Golgi complex at low temperatures (15°C) (Martinez-Alonso et al., 2005). Hibernation can lead to biochemical adjustments to the membrane composition, such as an increase in the levels of ceramides containing more than 20 C atoms, which reportedly contributes to GA instability (Fukunaga et al., 2000; Gonzalez-Riano et al., 2019).

Regarding protein synthesis reduction, electron microscopy studies in taste bud cells (Popov et al., 1999) and in CA3 pyramidal neurons (Bocharova et al., 2011) showed that torpor stages are associated with a transient reduction in the number of polyribosomes and in rough ER profiles. Regarding the neuronal GA, previous studies have reported transitory fragmentation or disassembly along with a reduction in the size of the GA and loss of flattened cisternae during torpor (Popov et al., 1999; Bocharova et al., 2011; Antón-Fernández et al., 2015). The present study adds the GA of neocortical glial cells (microglia, astrocytes and oligodendrocytes) to the list of brain structures undergoing morphological changes during mammalian hibernation.

A direct relationship between GA size and the level of cell activity has been established (Lucassen et al., 1993; Salehi et al., 1994). Therefore, the apparent fragmentation and size reduction of the GA observed in the present study during the torpor state – based on the expression of GA structural proteins (GM130, MG160, and Golgin84) – could be related to a reduced capacity of glial cells, in terms of protein processing, modification and targeting. Since the GA of the three glial cell types had alterations and each type has different functions, it is likely that the changes observed play different roles in the metabolic rate reduction that characterizes the hibernating process. Further electron microscopic studies are necessary to determine the extent of the Golgi ribbon fragmentation during hibernation in microglial cells, astrocytes and oligodendrocytes – to determine whether the changes observed in the present study might differentially affect the integrity of the Golgi stacks in the different glial cell lines.

In previous studies, we have reported that, during torpor, microglial cells show a dystrophic phenotype (Leon-Espinosa et al., 2018). Morphological changes of microglial cells during torpor are accompanied by a reduction in mRNA expression levels of the proinflammatory cytokine IL-1β (Cogut et al., 2017) and a lack of expression of the microglial cell activation markers CD16/32 and CD68 (Leon-Espinosa et al., 2018). In addition, previous studies demonstrated that during torpor the microglial reaction in the striatum against lesions induced by the insertion of microdialysis probes is dramatically reduced (Zhou et al., 2001). This wide range of plastic adaptations might prevent cerebral damage in hibernation, which has led to the proposal that hibernation is a natural model of neuroprotection (Zhou et al., 2001). We have shown that the GA of dystrophic microglial cells from torpid hamsters undergoes morphological alterations as seen by comparing the GA of euthermic microglial cells, with such alterations including the down-regulation of MG160 expression and fragmentation as revealed by immunostaining for GM130, MG160, and Golgin84. These changes suggest that the reduction and the fragmented appearance of the GA might be common phenomena affecting different cell types with reduced activity.

The morphological appearance of the GA of oligodendrocytes and astrocytes is also affected during the hibernation cycle of the Syrian hamster. The main function of oligodendrocytes is to generate the specialized membrane myelin sheath, which provides support and insulation to axons, allowing the rapid propagation of action potentials (Eshed-Eisenbach and Peles, 2013). Given that the GA contributes to myelin biogenesis, we suggest that the GA disorganization in oligodendrocytes – dispersion of Golgin84-ir elements through the perikaryal cytoplasm showing a sand-like appearance – could relate to a decrease in membrane biogenesis and polarization during torpor. In addition, the GA of protoplasmic astrocytes also undergoes plastic changes during the different phases of the hibernation cycle. We observed that, during torpor, MG160 staining was markedly reduced in the cytoplasm of astrocytes, but the expression of Golgin 84 was preserved, although the GA had a punctate or fragmented appearance, both in the soma and in the processes of astrocytes, as seen by comparing with the GA of astrocytes in euthermia. These changes might reflect a decrease in the activity of astrocytes concomitant with the general reduction of brain metabolism during torpor.

Finally, the return to normothermia from torpor that occurs during the arousal state has been related to neuroprotective roles (Daan et al., 1991; Arendt et al., 2003) and requires reorganization of cell organelles, synaptic hippocampal plasticity, Tau dephosphorylation, and recovery of normal microglial morphology (Arendt et al., 2003; Magarinos et al., 2006; Arendt and Bullmann, 2013; Antón-Fernández et al., 2015; Cogut et al., 2017; Leon-Espinosa et al., 2018). Here, we demonstrated rapid GA structural rebuilding and reorganization in glial cells during arousal (only 1 h after exiting torpor), as is also the case in the GA of neurons (Antón-Fernández et al., 2015). The up-regulation of Golgin84 expression during arousal (Antón-Fernández et al., 2015) may be required for the rapid rebuilding or reorganization of the GA during arousal in different neocortical cell types leading the animals from torpor to euthermia. In conclusion, the GA of glia undergoes significant structural changes along with differential modifications in distribution patterns of the GA structural proteins at different stages of the hibernation cycle. These changes might affect post-translational protein modifications as well as the sorting and trafficking of proteins in glial cells affecting their activity.

## Ethics Statement

All experimental procedures were carried out at the animal facility of the Instituto Cajal in Madrid (registration number ES 28079 0000184) and were approved by the Institutional Animal Experiment Ethics Committee (PROEX 292/15).

## Author Contributions

All authors listed have made a substantial, direct and intellectual contribution to the work, and approved it for publication.

## Conflict of Interest

The authors declare that the research was conducted in the absence of any commercial or financial relationships that could be construed as a potential conflict of interest.
